# 

**Published:** 1829-12

**Authors:** 


### MONTHLY LIST OF MEDICAL BOOKS.

[*Medical Works cannot be entered on this List except a copy be sent for the purpose; the titles of Books having frequently been transmitted to us, as published, which have not appeared for weeks, or even months, after.*]

A Supplement to Myology, illustrated by coloured Plates, on a peculiar Construction; containing the Arteries, Veins, Nerves, and Lymphatics, the Abdominal and Thoracic Viscera, the Brain, the Ear, the Eye, &c. By E. W. Tuson, F.L.S. Lecturer on Anatomy and Physiology, &c.—J. Wilson, London.

The present work is the supplement to one previously published on the Muscles, exhibiting them in layers, as they are seen in dissection. The intention of the author is to explain the course, situation, and relative position of the various parts mentioned in the title. The first plate, a dissection of the head and neck, exhibits at first sight the common integuments, which being elevated, shows the muscles, vessels, nerves, &c.; which can be raised, and the deeper-seated parts seen, until the bones are brought into view; by which means the connexion, course, and ramifications of the vessels are satisfactorily explained, giving an impressive idea of the structure of this part of the human body. The second plate contains the abdominal and thoracic viscera; and the following, the other important parts, on a similar construction.

The manner in which the work is contrived and executed, reflects infinite credit on the talent, industry, and perseverance of the author; and, in our opinion, it appears in every respect calculated to assist the study of that branch of science on which it treats. We can safely recommend it to the practitioner in the country, as likely to be of great practical utility in keeping up or refreshing his knowledge of the anatomical structure of the human frame. To the pupil, it will lay a foundation for his future studies; prepare and assist, in a great measure, his labours in the dissecting room; and to the non-professional man of science, its perusal will afford much pleasure and satisfaction.

Auli Cornelii Celsi De Re Medica; Libri Octo. Editio nova, ex recensione Leo Targa, curante C. F. Collier, M.D. Accedit Lexicon Celsianum breve. Vol. I. With an English Translation.—32mo. Highley, 1829.

A very useful assistant to the medical student who requires improvement in the Latin language.

An Introduction to Medical Botany. Illustrated with coloured Figures. By Thomas Castle, F.L.S. &c.—12mo. pp. 172. Cox, London.

The medical student, who cannot be expected to enter into the study of very elaborate works on medical botany, will find in this little work the elements of the subject explained with brevity and perspicuity.

*Communications have been received from* Dr. *and* Mr. Griffin, Dr. A. T. Thomson, *and* Mr. I. H. Burgess.

*The Editors are obliged to* Dr. Stroud *for his polite intimation.*

---

ERRATUM. At page 407, line 29, for "perspiration" read "respiration."

### INDEX

TO THE

SEVENTH VOLUME OF THE NEW SERIES

OF THE

### London Medical and Physical Journal.

PAGE

Abdomen, pain and tenderness of, after delivery, without peritoneal fever . . 66
Albumine, efficacy of, in case of poisoning by copper . . 132
Anasarca and ascites terminating suddenly and fatally . . 228
Anæsthesia, case of, (sensation lost, not motion) . . 76
Anatomy, Elements of General 449
Aneurism of brachial artery cured by pressure . . . 276
——— by anastomosis . 560
——— of subclavian artery . 371
———, temporal, from arteriotomy . . . 206
———, ligature applied at the *distal* side of . . . 371
Angina treated by lunar caustic 79
——— pectoris, pathology of . 547
———————, not always from ossification of coronary arteries 74
Ani, laceration of perineum and sphincter (cured by operation) 223
Animal magnetism . 115, 210, 315
Apoplexy without morbid appearances on dissection . . 439
Apothecaries, regulations of Society of . . . 472
——————— hall, regulations of 87
Arnott, J. M. esq. on Phlebitis . 137
Arterial system, extensive disease of . . . . . 129
——————————, general induration of . . . 367
Arteries, on ligatures of ruptured 180
Artery, wound of femoral, cured 566
Ascites, remarkable case of . 75
——— and anasarca, sudden and fatal termination of . . 228
Asphyxia, danger of insufflation in, 278
Asthma, occasional concomitants of, 71

PAGE

Asthma, metastasis of, into derangement . . . . 88

Bacot, Mr. on Syphilis . . 160
Belladonna, poisoning with . 468
Blisters, difficult to account for effects of . . . 78
Blood, on the buffy coat of . 400
Bones, reparation of fractured 454
Botanic garden at Chelsea, thrown open to medical students . 187
Brachial artery, aneurism of the, cured by pressure . . 276
Brain, life supported without, for two days . . . 555
——, symptoms of organic disease of . . . . 441
——, inflammation of membranes, not admitting of very active treatment . . . 434
——, effusion into ventricles, from rapid healing of extensive ulceration of leg . . 231
——, softening of . . 436
——, extensive extravasation of blood in . . . 440
——, pathology of . . 434
——, tubercular disease of . 438
Breast, case of irritable . 545
———, iodine in tumors of . 272
Brighton, climate of . . 335
Britain, superior salubrity of Great . . . 246
Brodie, B. C. esq. his opinion of Mesmerism . . . 214
Bubo, on pathology, causes, and treatment of . . . 1
Buffy coat of blood . . 400
Burns, compression in . . 492

Cæsarian section, cicatrization of womb after . . 82

PAGE

Calculus in the bladder, occasional difficulty in detecting . 204
Capillary vessels, physiology of 452
Carmichael's opinions on syphilis opposed by Mr. Bacot . 160
Carotid artery tied by Mr. Mayo, to arrest hemorrhage from ulcer in fauces . . . 510
Cataract, extraction of, by incision through upper part of cornea . . . 468
———, remarkable case of . 268
Cholera, in a school at Clapham 281
Chorea, cured by tartar-emetic ointment . . . 79
Civiale, M. operation for lithotrity, 276
Climate, on the influence of . 332
Colchicum, on vinous tincture of, 180
Combustion, on spontaneous human . . . . 280
Compression in phlebitis, &c. . 492
Conjunctiva, intermittent inflammation of . . . 515
Constantinople, medical practice in . . . . 374
Constipation, obstinate, cured by mechanical means . . 563
Contagion, on the prevention of the spreading of, in Gibraltar 88
Copper, poisoning by acetate of 132
Cornea, remedy for opacity of 83
———, case of sloughing ulcer of, treated by Argent. Nitr. . 128
Cramp of stomach, remarks on 177
Cranium, removal of portions of the bones of, in cases of disease 101
Croup, occasional existence of spasmodic . . . 70
———, treatment of . . 71
Cutaneous diseases, Dr. A. T. Thomson's edition of Bateman on . . . . 235
Cystocele, Mr. Burnett's case of 506
Cytisine (extract from laburnum) poisonous effects of . . 95

Death, perspiration after . 171
Devon, climate of south coast of, 336
Duplex child, account of a living, 459
——————— the death of . . . . 571
Dyspepsia, treatment of . 59
————, pathology of . 57

Earle, Mr. Henry, his opinion of mesmerism . . . 219
Ear, functions of different parts of the . . . 266
Eczema, Dr. A. T. Thomson differs from Bateman upon the subject of . . . 239

PAGE

Elliotson, Dr. his opinions on mesmerism . . . 315
Emphysema, case of general . 134
England, climate of south coast of . . . . 335
———————— west coast of 338
Ergot of rye, dangerous effects of, 181
————, effects of, as an aliment . . . . 278
Erysipelas, controlling power of, over other forms of disease . 223
————, compression in phlegmonous . . . 492
Eye, cases of diseases of . 328
—————, treated by Ol. Terebinth. &c. . 427, 521
——, influence of operations on the neck on the nutrition of . 266
Exhaustion in infants, resembling hydrencephalus . . 351
Extra-uterine life of child, proofs of . . . . 412

Face, on the nerves of . 525
Facial nerve, paralysis of, cured 40
Fauces, excessive hemorrhage from ulcer in; carotid artery tied by Mr. Mayo . . 510
Femoral artery, wound of, cured, 566
Fever, results of treating it by art or leaving it to nature . 244
—— hospital, Report of Dublin 171
——, Dr. O'Reardon's remarks on distinctions of . . ib.
Fœtation, case of extra-uterine 84
Foul drain producing cholera . 281
Foundling hospitals, statistics of 248
Fracture, compound, propriety of removing protruding extremities of bone in . . 27
————, seton successfully applied in disunited . . 275
Fractured bones, reparation of 454
France, climate of west and south-west of . . . 341
———————— south-east of 342
Fungous tumor of the lip . 327
Fungus hæmatodes, remarkable instance of constitutional nature of . . . 38

Garner, Mr. Robert, his examination for honours at the London University . . 184
Gas of burning coal, effects of inhalation of . . 557
Gastric juice, effects of, on stomach after death . . 365
Gastritis, acute, rare occurrence of . . . . 51
Genitals, malformation of . 517

PAGE
Genoa, climate of . . 345
Gonorrhœa, cure of . . 163
——— and syphilis, identity of . . . . . 161
Guthrie, G. J. esq. various cases of diseases of the eyes, 328, 427, 521

Hair, no canal within it . 263
Halford, Sir Henry, observations on insanity . . . 90
Hastings, climate of . . 335
Heart, no division between ventricles of . . . 264
———, polypus of . . 268
———, sympathetic affections of, how distinguished . 61
———, diagnosis of acute inflammation of . . . 176
———, state of, during pregnancy . . . 171
———, bone found in the . 367
Hectic fever, fallacy of the pulse in . . . . . 75
Hemorrhage arrested by contortion of arteries . . 463
——— after extraction of tooth . . . 559
———, hereditary tendency to 559
Hepatic duct, rupture of . 555
Hermaphrodism? case of . 517
Hernia, strangulated crural, with omentum in the sac . . 327
———, case of large scrotal, reduced . . . 515
———, strangulated; tumor neither tense nor inflamed . 199
——— cerebri, cured by sponge compress . . . 32
———, infrequent . . 504
Herpes zoster, severe cases of 569
Hip-joint, disease of . . 489
Holland, Dr. his opinion of mesmerism . . . 215
Hooping cough, treatment of, by quinine . . . 21
Hospitals, Medical Statistics of 247
Howship, Mr. his lectures announced . . . 379
Hydrencephalus, exhaustion in infants resembling . . 351
Hydriodate of potash, on adulteration of . . . 310
Hydrocyanic acid, as an external application in cutaneous diseases, 238
Hydrophobia, pustules on the tongue in . . 174, 462
——— treated by chlorine . . . . . 370
Hydrothorax, treated by lactuca sylvestris and digitalis . 176
———, case of . . 226

PAGE
Hysteria connected with functional disorders of the spinal cord . . . . 477
———, prussic acid in . 368
Hysteritis puerperalis, (Mr. Paxton's cases) . . 195

Ichthyosis simplex, interesting case of . . . 237
Iliac artery, rupture of the right common . . . 129
Impetigo, hydrocyanic acid as an application in cases of . 238
Inflammation destructive of action of muscular fibre . 50
———, the term, its limits 52
Inorganic and organic bodies distinguished . . . 451
Insanity, observations on, by Sir H. Halford . . . 90
———, effects of erysipelas on 225
Insects, sexual instincts of . 85
Iodine detected in the blood . 471
——— in mammary tumors . 272
———, on the use of . . 273
Irritation, death from mercurial 43
Iron, the hydrocyanate of, a substitute for quinine . . 369
Italy, climate of . . 345

Laburnum, injurious effects of the common . . . 86
———, poisonous properties of 93
Labour, affections of the viscera, joints, eye, &c. after . 149
Leech-bites, bleeding of, how stopped . . . 86
Leg, compound fracture of . 36
———, large tumor in calf of . 519
———, reunion of a large portion of the calf, torn off by accident 82
Leucorrhœa, metastasis of . 461
Life, phenomena of organic and animal . . . 549
———, influence of poverty, cold, and moisture upon . . 249
Ligatures, metallic, applied to arteries . . . 464
Lip, fungous tumor of . . 327
Lithotomy, Mr. Mayo's case of 425
———, success of the operation of . . . 83
——— *à deux temps* . 462
———, case of; stone behind the pubis . . 209
Lithotrity on a child æt. three years . . . 463
———, M. Civiale's operation 276
———, historical Sketch of, 285, 381
Liver, adhesion to, perforation of stomach existing . . 55

PAGE
London, climate of . . 334
———, comparative mortality of, much diminished latterly 245
Lunar caustic, in cases of angina 79
Lung, gangrene of the right . 135

Madeira, climate of . . 349
Magnetism, on animal 115, 210, 315
Male, influence of the, extending beyond a single impregnation 264
Malaria at Rome . . 347
Mamma, neuralgia of . . 545
Marseilles, climate of . . 343
Marriage, comparative fruitfulness of, in different countries 251
Mayo, Mr. H. his plan of treating varicose veins . . 208
—————, experiment of dividing the portio dura *on both sides* . . . . 530
Membrana caduca, on a cavity and fluid in . . . 264
Menstruation at the age of nineteen months . . . 366
————— during pregnancy 426
Mercurial irritation, death from 43
Mesmerism, Mr. Chenevix on, 115, 210, 315
—————, various opinions on ib.
Metallic ligatures applied to arteries . . . 464
Midwifery, Mr. Waller's Elements of Practical . . . 359
—————, Mr. Waller's half-yearly report of cases . 103
Morbid anatomy, specimens of, preserved . . . 568
Mortality the same in hospitals, under different modes of treating disease . . . 247
Montpelier, proportion of deaths at . . . . 246
—————, climate of . . 343
Morphine, effects of the acetate of, upon persons in health . 109
Mortality, decreasing ratio of . 245
Mucous membranes, structure and functions of . . 50

Naples, climate of . . 346
Nævi, cured by vaccination . 241
Nævus, becoming a large and bloody tumor . . ib.
——— cured by vaccination . 80
Nerve, tumor of radial . 431
Nerves of the face, on the . 525
Nervous tissue possesses the property of developing galvanic fluid . . . . 457
Neuralgic diseases, on . . 538
Neuralgia cured by colchicum 370

PAGE
Neuralgia, endermic use of quinia 272
Nice, climate of . . 344
Nitrate of silver in vaginal discharges . . . 306
Nymphomania, treatment of . 180

Ophthalmia, purulent, of infants, cases of, treated by Ung. Arg. Nit. 125
Opium eater, case of an . 86
Organic and inorganic bodies, differences between . . 451
Ovaria, case of scirrhous . 107
———, extirpation of diseased 462

Palsy cured by strychnia . 271
Pannus successfully excised . 180
Paralysis of the facial nerve cured 40
Paracentesis abdominis, little danger from operation of . 76
Parotid duct, fistula of, successfully treated . . . 133
Pau, climate of . . 342
Penis, cancer of, from phymosis 275
Penzance, climate of . . 337
Perspiration after death . 171
Perineum, important improvement in treating laceration of 466
—————, Mr. Mayo's successful operation for laceration of, and sphincter ani . . 222
Phlegmasia dolens, pathology of, by Dr. R. Lee . . 269
Phlebitis, compression in . 492
Phosphorus, death from taking 374
Phrenology, doctrines and facts of, opposed . . 253
—————, tracts on . . 252
Phthisis benefited at Rome . 347
Phymosis productive of cancer of penis . . . 275
Pisa, climate of . . 346
Placenta felt at the upper part of vagina, but not detached from uterus . . . 360
Plumbers, short life of . . 251
Polydipsia cured by camphor . 272
Polypus attached to fundus uteri, 556
Pregnancy, menstruation during 426
—————, state of heart during 171
Prout, Dr. N. his opinion of mesmerism . . . 214
Prussic acid, on the use of . 368
Pulse, fallacy of, in some cases of hectic fever . . . 75
Puerperal fever, epidemic . 64
—————, treatment of . 65
Pustule, compression curing malignant . . . 560

Quinine given as snuff . . 180

PAGE
Rectum, fistulous opening between the, and urethra, cured by actual cautery . 205
——, extirpation of a portion of 468
Respiration, on action of spinal marrow in . . . 554
Rome, climate of . . 346

Sarcocele, cured by bougies . 303
Scalp, tumor of, in new-born children . . . 106
Sciences, cultivation of, favorable to longevity . . . 250
Sea-sickness, prevention of . 471
Secale cornutum, dangerous effects of . . . 181
Semen, case of suppression of . 75
Serous membranes, morbid conditions of . . . 49
Sexes, relative numbers of the 251
Siamese double boys . . 569
Sidmouth, climate of . . 336
Sirvatan, on poison of . . 469
Skin, on the anatomy of . 263
Spasm of stomach, remarks on 177
Spiders, instinct of . . 85
Spinal cord, inflammation of the substance of . . . 442
————, functional disorders of 477
——— irritation, of . . 543
——— marrow, disease of, evinced by phenomena in remote parts . . . . . 539
————————, softening of . 368
Spine, fracture of . . 514
Statistics, on Medical . . 242
Stomach, pathology of . . 51
————, malformation of, in an infant . . . 458
————, on spasm or cramp of 177
————, cartilaginous degeneration of . . . 367
————, communication formed between arch of colon and . 55
————, perforation of . 174
————, ulceration of, not always productive of alarming symptoms . . . 53
Stricture, operation for impermeable . . . 325
Submersion, recovery after prolonged . . . 366
Sulphureous baths in lichen agrius, 236
Sulphuric acid (native) . 279
Summer residence, the most healthy . . . 348
Surgeons, regulations of Royal College of . . . 472
Surgical cases, Dr. Ballinghall's, 23, 95, 199
Syphilis, Mr. Bacot's treatise on 152

PAGE

Syphilis, on secondary . 167

————, non-mercurial treatment of . . . 157

———— and gonorrhœa, identity of . . . 161

Syphilitic sores, hardness generally a mark of . . 165

Tænia, male-fern root used to remove . . . 176
Tarantula, case of bite of . 557
Terebinthinæ, oleum, in diseases of the eye . . 328, 429
Testicle in groin, resembling hernia . . . 507
———, Mr. Guthrie's case of diseased . . . 426
Tetanus, Mr. Mayo's case of successful traumatic . . 220
————, singular treatment of 78
Thigh, luxation of, on pubes . 516
———, lacerated wound of . 34
Thorax, case of tumors in . 408
Tic Douloureux, case of, by the celebrated John Locke . 89
Tooth, hemorrhage after extraction of . . . 559
Torquay, climate of . . 336
Tracheotomy, successful case of 80
Trachea, morbid appearances on dissection . . . 69
Transfusion, on . . 369
———— of blood ; operation described . . . 361
Tubercles in the lungs, their nature . . . . 72
Twins, united by a bond of union . . . 569
Typhous fever, proofs of contagious nature of . . 12
——————, remarks on, in Halifax, &c. . . 11

Ulcer, new treatment of the deep and excavated . . 257
Umbilical vein inserted into the right auricle of the heart . 171
Urethra, fistulous opening between the, and the rectum, cured by actual cautery . 205
———, dilatation of female, more severe than operation for lithotomy . . . 202
Urine, suppression of, for seven weeks, cured . . 269
Uteri, removal of scirrhous os . 201
Uterus, muscularity of . 364
———, on irritability of . 68
———, case of excision of . 370
———, polypus attached to fundus of . . . 556

PAGE
Uterus, cancer of, cured by injections of hydrocyanic acid 274

Vaccination from the cow . 462
———— curing nævus 80, 241
Vaginal discharges, use of nitrate of silver in . . . . 306
Varicose veins, treated according to Mr. Mayo's plan . 207
Variola, on a fatal epidemic in Halifax, &c. . . . . 11
Variolaria amera, a substitute for quinine . . . 468
Varioloid disease, remarks on, in Halifax, &c. . . . 11
Variolous pustule, on the precise seat of . . . 189
Vascular system, anomalous formation of . . 170
Veins, pathological inquiry into secondary effects of inflammation of . . . 137
Veins, death in cases of inflammation of, not arising from its extension to the heart . 141
——, purulent effusion in various parts, in cases of inflammation of . . . 144
Vegetation in air at different pressures . . . 279

Warts, treatment of syphilitic 166
Westminster Ophthalmic Hospital, reports from, under direction of Mr. Guthrie, 328, 427, 521
Wight, climate of Isle of . 335
Womb, cicatrization of, after Cæsarian section . . 82
Women, important diseases of 64
———, mortality of, not greater at the *critical* period of life 250
Worari, on poison of . 469
Wounds in dissection, compression in . . . 492

### CRITICAL ANALYSES.

Pathological and Practical Researches on Diseases of the Stomach, the Intestinal Canal, the Liver, and other Viscera of the Abdomen. By John Abercrombie, m.d. . . . . . . . 47
An Account of some of the most important Diseases peculiar to Women. By Robert Gooch, m.d. . . . . . . . 63
An Account of the Morbid Appearances exhibited on Dissection in Disorders of the Trachea, Lungs, and Heart; with Pathological Observations, to which a Comparison of the Symptoms with the Morbid Changes has given rise. By Thomas Mills, m.d. . . . . . 69
A Pathological Inquiry into the Secondary Effects of Inflammation of the Veins. By J. M. Arnott, Surgeon . . . . 137
A Treatise on Syphilis. By John Bacot, Surgeon . . . 152
A Practical Synopsis of Cutaneous Diseases, by Thomas Bateman, m.d. Seventh Edition. Edited by A. T. Thomson, m.d. f.l.s. &c. . 235
Elements of Medical Statistics. By F. B. Hawkins, m.d. . . 242
Observations on the Phrenological Developement of Burke, Hare, &c. By Mr. Stone . . . . . . . . . 252
Answer to "Observations on the Phrenological Developement, &c." By Mr. Combe . . . . . . . . . ib.
A Rejoinder to the Answer of G. Combe, Esq. By Mr. Stone . ib.
Anti-Phrenology. By John Wayte, m.d. . . . . . ib.
Essay upon the Treatment of the Deep and Excavated Ulcer. By Mr. Stafford . . . . . . . . . 257
The Influence of Climate in the Prevention and Cure of Chronic Diseases. By James Clark, m.d. . . . . . . 332
On a Morbid Affection of Infancy arising from Circumstances of Exhaustion, but resembling Hydrencephalus. By Marshall Hall, m.d. 351
Elements of Practical Midwifery. By C. Waller, Esq. . . 359
Pathological and Practical Researches on Diseases of the Brain and Spinal Cord. By J. Abercrombie, m.d. . . . . . 434
Elements of General Anatomy. By R. D. Grainger, Esq. . . 448
On the Nerves of the Face. By Charles Bell, f.r.s. . . 525
A Treatise on Neuralgic Diseases. By Thomas Prigden Teale, Esq. 538
An Experimental Inquiry into the Laws which regulate the Phenomena of Organic and Animal Life. By G. Calvert Holland, m.d. . 549

### NAMES OF AUTHORS,

*Of whose Works, Observations, &c. either a detailed Account, or more or less general View, is given in the present Volume.*

PAGE

ABERCROMBIE, Dr. J. on Diseases of the Brain . . . 434
—————— analysis of his Pathological and Practical Researches on Diseases of the Stomach, &c. . . . . 47
ALBERT, Dr. case of Polydipsia cured by Camphor . . . 272
AMUSAT, M. on the Contortion of Bleeding Vessels to arrest Hemorrhage . . . . . . . . . 463
ARNOTT, Mr. J. M. analysis of his Pathological Inquiry into the secondary Effects of Inflammation of the Veins . . . . . 137
ARROWSMITH, Dr. on the Hydrostatic Test, and other Proofs of the Extra-uterine Life of the Child . . . . . . 412
BACOT, Mr. analysis of his Treatise on Syphilis . . . 152
BALLINGHALL, Dr. his Clinical Lecture . . 23, 95, 199
BARBIER, Dr. case of Bone found in the Heart . . . 367
BARDSLEY, Dr. remarkable case of Ascites . . . . 75
BARTHELEMY, Mr. on Softening of Spinal Marrow . . . 368
BAYLE, Dr. on Iodine in Mammary Tumors . . . . 272
BELL, CHARLES, F.R.S. on the Nerves . . . . . . 525
BECLARD, M. case of Fistula of the Parotid Gland and Duct, successfully treated . . . . . . . 133
BENNERSCHEIDT, M. on Iodine detected in the Blood . . 471
BERAUDI, Dr. L. on the Galvanic Properties of the Nervous Tissue . 457
—————— on Effects of Morphine upon Persons in Health . 109
BLAKE, Dr. on the Buffy Coat of the Blood . . . . 400
BOIVIN, Madame, on Muscularity of the Uterus . . . 365
BORLAND, Dr. account of a living Duplex Child . . . 459
BRESCHET, M. on a Cavity in the Membrana Caduca . . . 264
BRIGHT, Dr. case of Malformation of Genitals . . . 517
BRODIE, B. C. esq. case of Lacerated Wound of the Thigh . . 34
—————— case of Compound Fracture of the Leg . . . 36
BROSIUS, Mr. on treatment of Hydrothorax . . . . 176
BROUGHTON, S. D. esq. on Bubo . . . . . . 1
BRUNI, Dr. on Cancer of the Uterus cured by Prussic Acid . . 274
BURNETT, Mr. G. T. cases of infrequent Hernia . . . 504
CAMPAGNO, Dr. case of Neuralgia Facialis . . . . 370
CASSEBEER, M. on Variolaria Amera as a Substitute for Quinquinia . 468
CAUCANAS, Dr. on Endermic Method of using the Sulphate of Quinia 272
CHELIUS, Professor, on Colchicum . . . . . 180
CHENEVIX, R. esq. on Mesmerism . . . 114, 210, 315
CIVIALE, M. operation of Lithotrity . . . . . 276
CLARK, Dr. JAMES, on Influence of Climates . . . . 332
COMBE, Mr. GEORGE, answer to Mr. Stone . . . . 252
COSTELLO, W. B. esq. Historical Sketch of Lithotrity . . 285, 381
COURHAUT, M. on Ergot of Rye . . . . . . 278
DAVIES, Mr. J. H. on Sexual Instinct of Insects . . . 85
DERBYSHIRE, Mr. remedy for Sea-sickness . . . . 471
DEWEES, Dr. Work announced . . . . . . . 87
DE FONTENELLE, M. JULIA, on Spontaneous Combustion . . 280
DE RACUM, Dr. case of Suppression of Urine for seven weeks . 269
DIEFFENBACH, Dr. on Transfusion . . . . . . 369
—————— improved method of treating Laceration of the Perineum 466
—————— case of Cartilaginous Degeneration of the Stomach . 367

PAGE
DOBEREINER, M. on Vegetation . . . . . . 279
DOHLDORF, Dr. case of Seton successfully applied in disunited Fracture 275
DONELLY, Dr. on Typhus and Variola . . . . . . 11
DUPUYTREN, M. case of Aneurism of Subclavian Artery . . 371
——— on Ligatures of Ruptured Arteries . . . 180
——— case of Paralysis of the Facial Nerve . . 40
EARLE, H. esq. case of large Scrotal Hernia . . . . 515
EATON, Professor, on native Sulphuric Acid . . . . 279
EBERS, Dr. J. J. on removal of Tænia by Fern root . . . 176
ESSER, Dr. C. L. on the Functions of the Ear . . . . 266
FAHNESTOCH, Dr. W. M. on Iodine . . . . . . 273
FALLOT, Dr. case of intermittent Inflammation of Conjunctiva . 515
FRANCHE, Professor, case of Anomaly of the Vascular System . . 170
FUCHS, Dr. F. on Pustules and Vesicles on the Tongue in Hydrophobia 462
GARNER, Mr. ROBERT, his examination for honours at the University of London . . . . . . . . . 184
GIBBS, Dr. H. L. case of Tumor of the Radial Nerve . . . 431
GRAEFE, Professor, case of Pannus cured . . . . 180
———, J. V. on Cataract . . . . . . 468
GRAINGER, Mr. R. D. on General Anatomy . . . . 448
GRIFFIN, Dr. W. on Functional Disorders of the Spinal Cord . . 477
———, Mr. D. on Functional Disorders of the Spinal Cord . . ib.
GOOCH, Dr. analysis of his Account of some of the most important Diseases of Women . . . . . . . . 63
GRÖSCHNER, Dr. case of Reunion of a large portion of the Calf of the Leg, 82
GUTHRIE, G. J. F.R.S. on the use of the Ol. Terebinth. in Ophthalmia . 329
——— cases of Purulent Ophthalmia of Infants (reported by Mr. FOOTE) . . . . . . . 125
——— case of Diseased Testicle . . . 426
——— cases from the Royal Westminster Ophthalmic Hospital 427
——— case of large Tumor in the Calf of the Leg . . 519
——— Report of Cases at the Royal Westminster Ophthalmic Hospital . . . . . . . . 520
HALFORD, Sir HENRY, on Insanity . . . . . 90
HALL, Dr. MARSHALL, on Perforation of the Stomach and Œsophagus 174
——— on Exhaustion in Infancy resembling Hydrencephalus 351
HANCOCK, Mr. on the Worari and Sirvatan . . . . 469
HARROWER, Dr. on Chorea . . . . . . . 79
HASSE, Dr. on Hydrocyanate of Iron as a Substitute for Quinine . 369
HAYWARD, Dr. on Prussic Acid . . . . . 368
HAWKINS, Mr. F. B. analysis of Medical Statistics . . . 242
HEBERDEN, Dr. on the Effects of Blisters . . . . 78
——— on the Fallacy of the Pulse in Hectic Fever . . 75
HEIM, Dr. on Diagnosis of Inflammation of the Heart . . . 176
HEMING, Mr. G. O. on the Seat of Variolous Pustule . . . 189
HEUSTIS, Dr. his case of Hernia Cerebri . . . . 32
HODSON, T. esq. case of Lithotomy . . . . . 209
HOLLAND, Dr. C. on the Laws of Organic and Animal Life . . 549
HOPFER, Dr. cases of Extirpation of Diseased Ovaria . . 462
HOWE, Dr. Z. case of Tracheotomy . . . . . 80
HOWSHIP, Mr. Surgical Lectures announced . . . . 379
HUSSON, M. on Pustules on the Tongue in Hydrophobia . . 174
HUSTON, Dr. R. W. on dangerous Effects of Secale Cornutum . . 181
JACKSON, Dr. SAMUEL, case of Gangrene of Lungs . . . 135
———, case of Fever, with Death, from Mercurial Irritation 43
JAHN, Dr. case of general Emphysema . . . . . 134
JEFFERY, Mr. on preventing the Spreading of Contagion in Gibraltar . 88
JEWEL, G. esq. on Vaginal Discharges . . . . . 306
LARCHER, Mr. on the State of the Heart during Pregnancy . . 171
LEE, Dr. R. case of Phlegmasia Dolens . . . . 269

PAGE
LEONARD, Mr. J. case of Scirrhous Ovaria . . . . . 107
LEVERT, M. on Metallic Ligatures . . . . . 464
LEROY, M. on treatment of Asphyxia . . . . . . 278
LISFRANC, M. case of Extirpation of a portion of the Rectum . . 468
LIZARS, Mr. remarks on the Operation of Lithotomy *à deux temps* . 462
LOCKE, JOHN (the celebrated), case of Tic Douloureux . . 89
LÖWENHARDT, Dr. on stopping Bleeding from Leeches . . 86
MACFARLANE, Dr. J. on Spasm or Cramp of Stomach . . . 177
MADDEN, R. R. esq. on the Medical Practice in Constantinople . 374
MAYER, M. Influence on the Eye from Operations in the Neck . 266
MAYER, Professor, on Cicatrization of the Womb after the Cæsarian Section . . . . . . . . . . 82
MAYO, HERBERT, F.R.S. case of Lithotomy . . . . . 425
——— case of Menstruation during Pregnancy . 426
——— case of Hemorrhage from Ulcer in Fauces; Ligature of the common Carotid . . . . . 509
——— case of Traumatic Tetanus successfully treated 220
——— case of Laceration of the Perineum and Sphincter Ani; Operation successful . . . . . . 222
——— successful Operation in a case of Impermeable Stricture . . . . . . . . 325
——— case of Strangulated Crural Hernia . . . 327
——— removal of a fungous Tumor from the Lip . ib.
MENDE, Professor, case of Insertion of Umbilical Vein in the right Auricle of the Heart . . . . . . . 171
MILLS, Dr. analysis of his Account of the Morbid Appearances in Disorders of the Trachea, Lungs and Heart, &c. . . . 69
NORTH, JOHN, F.L.S. case of Sarcocele . . . . . 303
OZANAM, Dr. treatment of Nymphomania . . . . 180
PAGENSTECHER, Dr. case of Malformation . . . . 458
PAXTON, Mr. JAMES, cases of Hysteritis Puerperalis . . . 195
PEREIRA, J. F.L.S. on Adulteration of Hydriodate of Potash . . 310
POMMER, Dr. on Digestion of the Stomach after Death . . 365
O'REARDON, Dr. case of Extra-uterine Fœtation . . . 84
——— Medical Report of Fever Hospital, Dublin . . 171
RECAMIER, M. case of Excision of the Uterus . . . . . 370
RÉNÉ BOURGEOIS, M. case of Recovery after prolonged Submersion . 366
REID, Dr. W. on singular treatment of Traumatic Tetanus . . 78
REID, A. esq. case of Anæthesia . . . . . . . 76
RENNER, Professor, case of Cyst near the Parotid Gland, containing a Fœtus . . . . . . . . . . 171
RICHET, Dr. P. on Quinquina taken as Snuff . . . . . 180
RIGACCI, M. case of Polypus of the Heart . . . . . 268
ROUX, M. on Cancer of Penis from Phymosis . . . . . 274
SCHOENBERG, M. on treatment of Hydrophobia . . . 370
SCHUTTE, Dr. case of Suppression of Semen . . . . . 75
SEGALAS, M. Lithotrity performed on a Child . . . . . 463
SEMMOLA, M. on treatment of Hydrophobia . . . . . 370
SPERANZA, M. on Perspiration after Death . . . . . 171
STAFFORD, Mr. R. A. on Deep and Excavated Ulcer (analysis) . 257
STONE, Mr. on Phrenology . . . . . . . 252
——— Rejoinder to Mr. Combe's Answer . . . . . ib.
TEALE, Mr. T. P. on Neuralgic Diseases . . . . . 538
TETU, M. Aneurism of Brachial Artery cured by Pressure . . 276
THOMAS, Mr. W. J. case of Diseased Hip-joint . . . 489
THOMSON, Dr. A. T. on Poison of Laburnum . . . . . 93
——— analysis of his edition of Bateman's Synopsis of Cutaneous Diseases . . . . . . . 235
TOIRAC, M. on the application of Lunar Caustic in Angina . . 79

| | PAGE |
|---|---|
| TURNER, Dr. on Metastasis of Asthma into Derangment | 88 |
| VELPEAU, M. on Compression in Phlebitis, &c. | 492 |
| VINCENT, Mr. case of Luxation of the Thigh-bone | 516 |
| VIRICEL, M. J. M. great Success in Lithotomy | 83 |
| WALLER, C. esq. Midwifery Report | 103 |
| ——— on Practical Midwifery | 359 |
| WASSENFUHR, M. case of Metastasis of Leucorrhœa | 461 |
| WAYTE, Dr. JOHN, notice of his work "Anti-Phrenology" | 252 |
| WEBER, Dr. on the Skin | 263 |
| WEBER, Professor, on the Instinct of Spiders | 85 |
| WENDELSTROM, Dr. curious case of Cataract | 268 |
| WHITE, Mr. case of Impermeable Stricture | 325 |
| WITTCHKE, Dr. case of Malformation of the Heart | 264 |
| WRIGHT, Dr. cases of Erysipelas | 223 |
| ——— case of Hydrothorax | 226 |
| ——— case of Anasarca and Ascites | 228 |
| ——— case of extensive Ulceration of Leg | 231 |

| | |
|---|---|
| COLLECTANEA | *Pages* 75, 170, 263, 364, 457, 554 |
| INTELLIGENCE | 86, 184, 281, 378, 471, 569 |
| METEOROLOGICAL REGISTER | 92, 188, 284, 383, 476, 572 |

### PLATES.

| | |
|---|---|
| Lithotritic Instruments | 381 |
| Mr. MAYO's Case of Ulcer in the Fauces | 513 |

J. and C. Adlard, Printers, Bartholomew Close.

